# Sound Wave Energy Resulting from the Impact of Water Drops on the Soil Surface

**DOI:** 10.1371/journal.pone.0158472

**Published:** 2016-07-07

**Authors:** Magdalena Ryżak, Andrzej Bieganowski, Tomasz Korbiel

**Affiliations:** 1 Institute of Agrophysics, Polish Academy of Sciences, Doświadczalna 4, 20–290 Lublin, Poland; 2 AGH University of Science and Technology, Department of Mechanics and Vibroacoustics, Krakow, Poland; Technical University of Madrid, SPAIN

## Abstract

The splashing of water drops on a soil surface is the first step of water erosion. There have been many investigations into splashing–most are based on recording and analysing images taken with high-speed cameras, or measuring the mass of the soil moved by splashing. Here, we present a new aspect of the splash phenomenon’s characterization the measurement of the sound pressure level and the sound energy of the wave that propagates in the air. The measurements were carried out for 10 consecutive water drop impacts on the soil surface. Three soils were tested (*Endogleyic Umbrisol*, *Fluvic Endogleyic Cambisol* and *Haplic Chernozem*) with four initial moisture levels (pressure heads: 0.1 kPa, 1 kPa, 3.16 kPa and 16 kPa). We found that the values of the sound pressure and sound wave energy were dependent on the particle size distribution of the soil, less dependent on the initial pressure head, and practically the same for subsequent water drops (from the first to the tenth drop). The highest sound pressure level (and the greatest variability) was for *Endogleyic Umbrisol*, which had the highest sand fraction content. The sound pressure for this soil increased from 29 dB to 42 dB with the next incidence of drops falling on the sample The smallest (and the lowest variability) was for *Fluvic Endogleyic Cambisol* which had the highest clay fraction. For all experiments the sound pressure level ranged from ~27 to ~42 dB and the energy emitted in the form of sound waves was within the range of 0.14 μJ to 5.26 μJ. This was from 0.03 to 1.07% of the energy of the incident drops.

## Introduction

Soil is a very important element and is the basis of many ecosystems; therefore, protecting it and identifying the factors causing its degradation have been a topic of scientific research for some years. Water, the basis of life on Earth, may in certain circumstances contribute to environmental degradation, for example water erosion [1–4].

The first phase of water erosion is the splash, when a drop of water (rainfall) hits the soil surface [5]. Research into the splash phenomenon has been underway for many years, since a complete description is very important from the point of view of modelling water erosion in the context of eliminating this negative phenomenon, both for agriculture as well as for the safety of people (e.g., preventing mudslides).

The phenomenon of the splash is examined using different techniques, both in the field and in laboratory conditions. Most are based on measuring the weight of the transported material [6–9]. To estimate the weight, various type of erosion cups were used in field conditions [10–13], while for the measurements carried out in the laboratory the appropriate sets of trays for collecting splashed particles were used [5,7,14].

Part of the research has focused on the impact of individual droplets on the ground [15,16]. Such studies were conducted mostly in laboratory conditions because it is easier to dose individual drops and observe the phenomena occurring on the surface of the soil in a controlled manner. As a result of such measurements, the following has been determined, (among other things): (i) the splash weight and matrix potential of a soil sample as well as the soil strength [17]; (ii) the splash distance and soil shear strength, the particle size and the thickness of the water film on the surface of the sample [10]; and (iii) the soil crusting and splash magnitude [18]. Using the image analysis method, the splash phenomenon was described as a result of the fall of a single drop, which was previously not possible due to the mass of the splashed material being too small [19].

The splash phenomenon occurring when a droplet impacts a surface is so short that its precise observation requires the use of so-called ‘high-speed’ cameras. Thanks to the use of images from such cameras, the relationship between the following were determined: splash angles and the soil shear strength [20]; the analysis of the shape and rate of formation of the splash and corona walls depending on the surface on which it took place [21]; the phenomena connected with the crater formation process [22]; the description of the splashes of dry sand [23]; and the initial soil moisture and splash reproducibility [24]. The use of high-speed cameras with modern software also allows the determination of the trajectory of the particles detached by a splash [25].

In addition to research focusing on the weight of the transferred material or the observation and description of the same phenomenon, attempts have also been made to estimate the energy required to initiate rainfall splash phenomena. Sharma and Gupta [6]—determined the threshold energy of splashes for sand samples with a different pressure head (which is a form of expression of the binding energy of water through the soil matrix in other words, this magnitude informs about the water content). For a nearly saturated sample (ψ = 0.1 kPa), this value was near zero, while for ψ = 1.5 kPa the value was about 0.07 mJ, which corresponds to 0 to 10% of terminal kinetic energy for a 4.6 mm drop. Salles et al. [12] confirmed the limit values (threshold energy) that falling drops needed so that the splash phenomenon would occur—these values were 5 μJ for sand and 12 μJ for silt loam [12]. Attempts were also made to estimate the energy associated with the different phenomena occurring as splashes. Ghadiri and Payne [21], used images from a high-speed camera and, estimated that between 10 and 25% of the kinetic energy of the raindrop becomes the kinetic energy of splash droplets. Additionally, Ghadiri showed that the crater absorbs 13 to 23% of the energy of falling drops [22]. Ahn et al. [26] reported that, for research on glass beads, 0.26% of falling drops’ energy was used for displacing of particles at a distance greater than 1 cm in the case of hydrophilic beads (3.1 * 10^−7^ J) and 0.78% (9.3 * 10^−7^ J) in the case of hydrophobic beads. However, studies of the energy balance of the splash phenomenon are still relatively modest and fragmented. Among the research, there are ideas for the analysis of information from the sound effects accompanying striking of drops of water on water or solid surfaces. Kinnell [27] recorded water droplet and glass bead impacts on a sensor recording the frequency of oscillation. Pumphrey and Walton [28] described the results from an experiment in which the sound emitted by water drops impacting on a water surface were recorded. The history of research using the noise of rain was presented by Prosperetti and Oguz [29]. However, the majority of their research was related to underwater noise and focused on the sound coming from falling water drops on water. It is difficult to find publications describing the testing of the acoustic wave that accompanies a splash when a drop of water hits the soil’s surface.

The aim of this study was to determine the sound pressure level and related energy of the sound wave dissipated during the splash propagating in the air. This energy was determined by measuring the sound pressure wave caused by a drop of water hitting the soil’s surface. These studies are one of the elements necessary to determine the energy balance of the splash phenomenon.

## Materials and Methods

The research was conducted using representative Polish soils (*Endogleyic Umbrisol*, *Fluvic Endogleyic Cambisol* and *Haplic Chernozem*) from an arable layer [30] (Table 1). The authors declare that no specific permissions were required for the locations shown in Table 1 and confirm that the field studies did not involve endangered or protected species.

**Table 1:** Particle size distributions of the soils used for the tests and their initial moisture content.

Soil		Particle size distribution* (%, diameter mm)			Initial water content	
Type /Location	Granulometric group	Sand 2–0.05	Silt 0.05–0.002	Clay <0.002	Pressure head [kPa]	Moisture (v %)
					0.1	27.8
*Endogleyic Umbrisol (Arenic)/* Boruja Nowa, Poland	sand	85.33	13.80	0.87	1.0	26.5
					3.16	24
					16	12
					0.1	21.6
*Fluvic Endogleyic Cambisol (Siltic)*/ Janówka, Poland	silt loam	23.10	67.06	9.83	1.0	21
					3.16	19.8
					16	18.7
					0.1	29.7
*Haplic Chernozem (Anthric)*/ Ulhówek, Poland	sandy loam	59.74	25.85	4.17	1.0	28.6
					3.16	27.6
					16	25

* Measured by a laser diffraction method, the same procedure as in Polakowski et al. [31, 32].

The samples were dried and sieved (2 mm mesh), placed in aluminium rings (1 cm high and 4 cm in diameter), secured with a chiffon cloth at the bottom to prevent soil loss, then humidified to four initial moisture contents (pressure head: 0.1, 1.0, 3.16 and 16 kPa). The criterion for soil selection was to differentiate their properties. This is because the particle size distribution seems to be most important in the context of the planned investigations into sand, silt loam and sandy loam–representative of Poland—which were sampled.

In order to unify the initial conditions for all the soil, the same water pressure heads were prepared. The particle size distributions, the investigated water pressure heads and the corresponding moisture content are presented in Table 1.

The measuring system was installed in the anechoic chamber of the AGH University of Science and Technology (Krakow, Poland). It consisted of a droplet forming and a recording system. The formation system dosed droplets 4.279 ± 0.002 mm in diameter, with a frequency of approx. 30 per minute. The drops fell from a height of 1.5 m. The kinetic energy of the drops was 0.491 mJ ± 0.012 mJ. The sound accompanying the water drops hitting the surface of the soil was recorded using a multi-channel microphone system (S1 Fig). For data acquisition, two NI-9234 measuring cards were used which enabled measurements with a sampling rate of 51,200 Hz and a resolution of 24 bits.

Since the aim of the research was to determine the energy of the sound wave propagating in the air, assuming the axial symmetry phenomenon (the vertical axis), eight microphones were arranged on one plane at a distance of 1 m from the source (the ring containing the soil). The measuring track was built from a set of eight microphones of type 40PH, with the output ICP, from G.R.A.S. Sound & Vibration Company (Denmark). These microphones have a frequency range of 10 Hz to 50 kHz with an accuracy of ±3 dB, a sensitivity of 50 mV/Pa and a diameter measuring one-quarter of an inch. Assuming axial symmetry of the phenomenon (on the vertical axis) microphones were arranged in one plane, at a distance of 1 m from the place of incidence of the drops.

The experiment was implemented through registering sound pressure changes caused by 10 consecutive drops falling on the surface of the same sample. All the measurements were done in 15 replicates, each prepared in the same manner. To conduct the research and registration of the sound pressure, a dedicated application software measurement using LabView was developed. On detection of hitting, the system recorded the sound for a strictly limited period of time (300 ms), while the first 50 ms was sometimes a pre-sampling, i.e., the time before the moment of impact detection of the drops. By such programming, the system could obtain a complete characterization of the phenomenon. Both the entire measurement time and the pre-sampling time were chosen experimentally. The impact detection method was based on the conjunction of events, frequency and amplitude. The trigger thresholds were chosen experimentally.

### Calculations

The measured parameter was the sound pressure level (mean value from eight microphones) at a certain distance (1 m) from the sound source. The sound pressure level *L*_*p*_ (expressed in decibels [dB]) is the logarithm of the pressure ratio causing the sound *p* to the reference pressure *p*_*0*_, defined by the formula:

$$L_{p}=10\;\log_{10}\left(\frac{p_{}^{2}}{p_{0}^{2}}\right)$$ (1)

where the reference pressure *p*_*0*_ is a contact sound pressure that causes a human hearing impression and has a value of 2·10^−5^ Pa. After the transformation of Formula 1, it was possible to calculate the pressure *p*.

The energy corresponding to the registered sound wave was determined from the formula:

$$E=\frac{N}{t},$$ (2)

where:

t–time [s],

N–an acoustic power of sound source equals

$$N=\frac{p^{2}}{Z}S\left[W\right],$$ (3)

p–acoustic pressure [Pa],

Z—medium wave impedance [N s/m^2^] for air at T = 20°C, and pressure 760 mmHg Z_0_ = 412 [N s/m^3^], and

S—the surface area of measuring sphere S_sphere_ = 4πr^3^ = 12,6[m^2^].

The impact of rain drops on the ground creates sound pulses. To determine the equivalent sound level based on other analyses, the assumed time of the incident was equal to 40 ms. At that time, the energy of the acoustic wave expressed on the basis of Formulas 2 and 3 was averaged as:

$$E=\frac{p^{2}S}{Zt}.$$ (4)

## Results

### Sound characteristics

**Fig 1:**
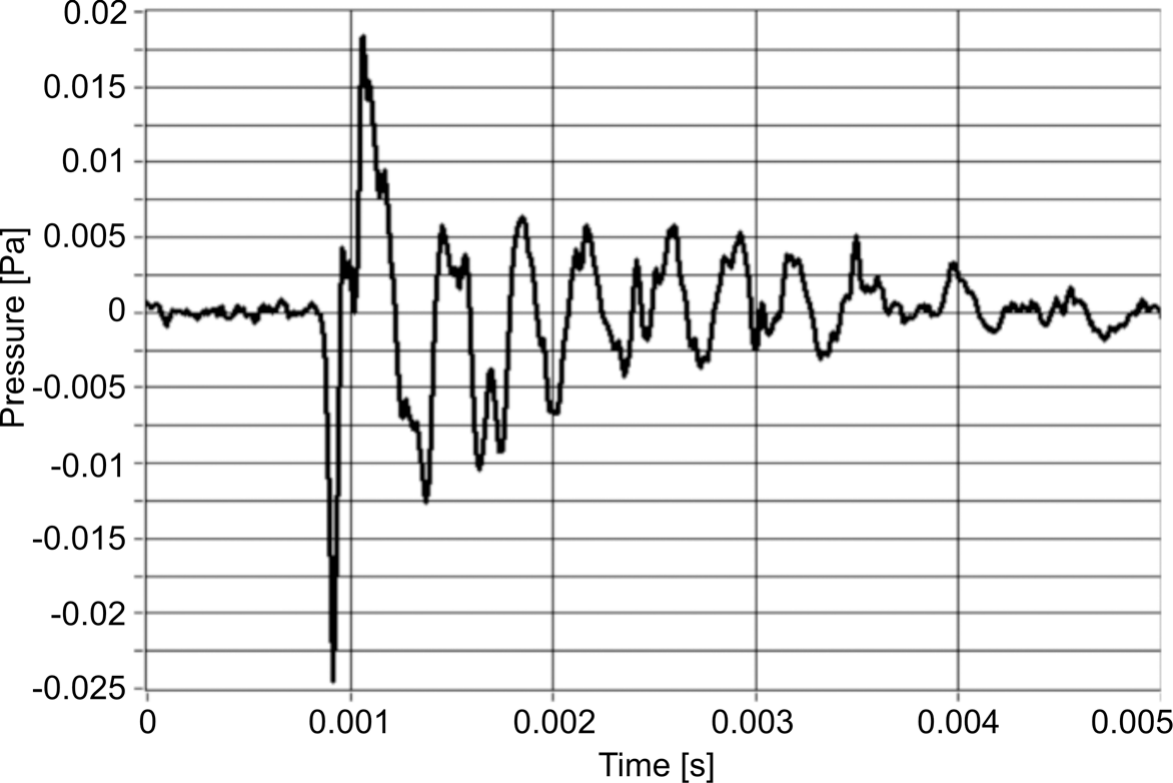
Example of the timing signal for *Endogleyic Umbrisol* and a pressure head of 3.16 kPa after 10 drops. Characteristics recorded on one of the eight microphones.

The signal of the instantaneous value of the sound pressure generated by water droplets striking the ground constitutes a non-stationary course of a polyharmonic, fading character (Fig 1). The peak value of the signal, depending on the ground, was from 3*10^−4^ Pa (for *Fluvic Endogleyic Cambisol* with a pressure head of 16 kPa) to 5*10^−3^ Pa (*Endogleyic Umbrisol* with a pressure head of 0.1 kPa). The meansquare value of the signal normalized to 40 ms was from 65*10^−5^ Pa (for *Fluvic Endogleyic Cambisol* and a pressure head of 16 kPa) to 25*10^−4^ Pa (for *Endogleyic Umbrisol* and a pressure head of 0.1 kPa). The maximum time of put-out of the signal equal to 36 ms was obtained for *Endogleyic Umbrisol* with a pressure head of 3.16 kPa.

### Sound pressure level (noise) of falling drops

**Fig 2:**
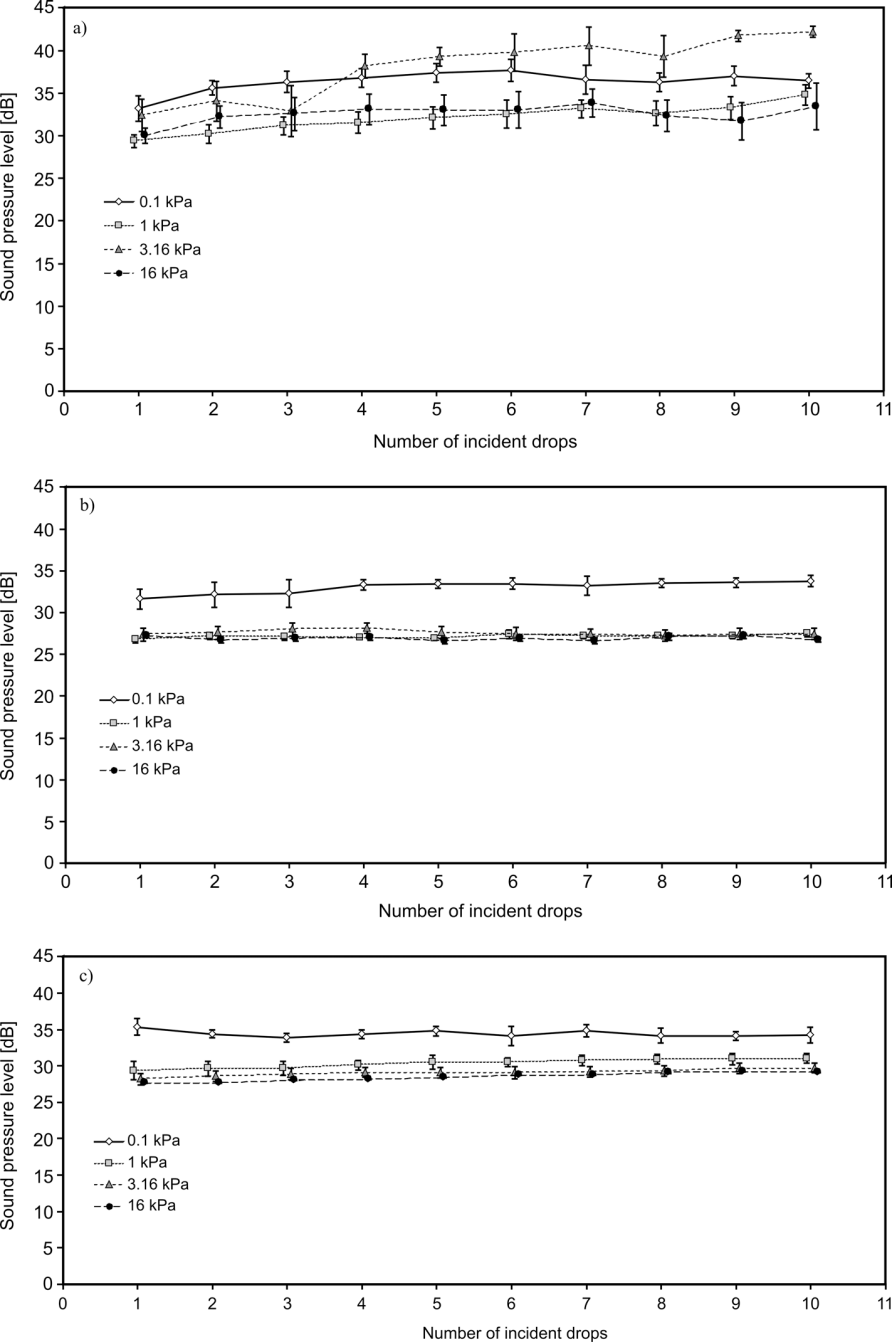
**Sound pressure level at the time of a drop of water hitting the surface of the soils studied for different initial moisture contents for a) *Endogleyic Umbrisol*, b) *Fluvic Endogleyic Cambisol*, c) *Haplic Chernozem***.

The sound pressure levels of the accompanying the impact of the water drops on the soil surface for all the tested soils with different initial values of soil water potentials (pressure heads) are shown in Fig 2 and S1 Table.

The analysis of the data from Fig 2 and S1 Table leads to the conclusion that both the highest noise value and the greatest variability were observed for *Endogleyic Umbrisol*. This soil is characterized by the highest content of sand fraction (85%). Among the four investigated humidities, the greatest diversity was observed with a soil water potential ratio of 3.16 kPa. In this case, it increased from 32 ± 2 dB to 42 ± 1 dB, with the lowest noise recorded for the first and the highest for the 10^th^ drop falling in the same place.

The least noise accompanied drops of water hitting the surface of *Fluvic Endogleyic Cambisol* (an average of about 28.5 dB). The highest occurred for *Endogleyic Umbrisol* (an average of about 34 dB). In the case of *Haplic Chernozem*, this amounted to an average of about 30.5 dB. The highest and most varied sound pressure levels occurred for soil containing the highest sand fraction; the smallest and least variable were for soils containing the highest fine fractions (silt and clay). For water hitting water, the sound pressure level was 30 dB.

### Energy sound waves emitted by successive drops for different soils

**Fig 3:**
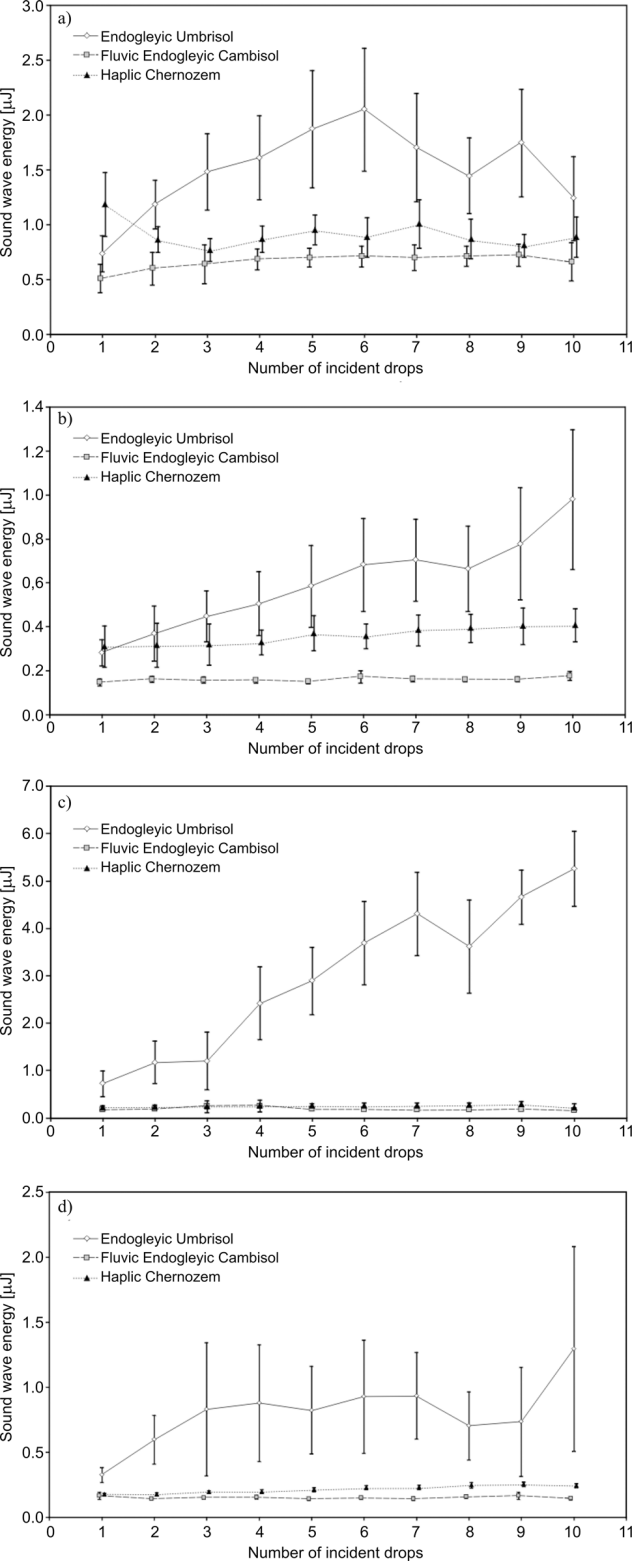
**Sound wave energy propagating in the air, emitted during the fall of 10 consecutive drops for different initial pressure head: a) 0.1 kPa; b) 1 kPa; c) 3.16 kPa; d) 16 kPa.** The indicated intervals represent the standard deviation.

The energy values of the sound waves emitted during the impact of 10 consecutive droplets on the soil surface studied at different initial pressure head are shown in Fig 3 and S2 Table. To show whether the differences shown in Fig 3 are statistically significant, the median test was carried out. The results of this test are included in the supplement S3 Table.

The first observation that arises after analysing the data of Fig 3 is the significantly higher values of energy obtained for *Endogleyic Umbrisol*. For the other soils the wave energy emitted for *Haplic Chernozem* greater than that for *Fluvic Endogleyic Cambisol*.

Considering the amounts of wave energy emitted by the successive drops once again the results for *Endogleyic Umbrisol* stand out from the rest. For this soil and, for all initial pressure heads, a significant increase of energy for the first few drops was visible. Then for all water contents the local maximum occurred (after the sixth or seventh water drop) and after the small decreases, further increases were evident (apart from with pressure head 0.1 kPa where the energy of the last drop was again lower). For both remaining soils it can be accepted that there were no trends and the values were similar for all successive drops; however, the energies for *Haplic Chernozem* were generally slightly higher than for *Fluvic Endogleyic Cambisol*.

## Discussion

### The influence of the particle size distribution on sound wave energy

The first aspect, that should be discussed is the comparison of the obtained results for investigated soils. It can be seen in Fig 3 that in all cases the highest values of sound energy were obtained for *Endogleyic Umbrisol*. It should be emphasized that the trend is the same for sound pressure because the calculation of sound energy is based on mathematical calculations where the sound pressure is the main input data (see the Calculations section).This tendency was the case regardless of the initial pressure head.

From the two remaining soils the higher values of energy (and sound pressure) were obtained for *Haplic Chernozem*–the differences were larger for soils containing more moisture (Fig 3A and 3B) and smaller for drier soils (Fig 3C and 3D).

The explanation of the differences in wave energies from different soils is the particle size distribution (Table 1). It can be stated that a larger amount of sand and smaller amount of clay leads to a higher energy sound wave. Similarly, the sandier the soil, the higher the sound waves pressure of the wave emitted after a drop of water hit the soil surface.

The reasons for this situation are varied. First of all, the sandier the soil, the looser it is (less compact) [33]. Hitting a less compacted soil surface (such as *Endogleyic Umbrisol*) causes greater movement of soil particles. The higher the humidity, the easier is to form craters—particularly in sandy soils [12]. It is precisely this movement of particles relative to each other that is an important factor in forming a sound wave. The confirmation of the correctness of such an argument comprises the lowest energy values obtained for *Fluvic Endogleyic Cambisol*. This soil contains a large number of fine particles and exhibited significantly less sensitivity to splashing (from droplet impacts on the surface becoming detached individual soil particles) and the formation of craters (practically not observed) for any initial pressure head.

The mentioned about creation of the crater can be the explanation of the decreasing of the sound wave energy for highest water content (0.1 kPa) *Endogleyic Umbrisol* after the 6^th^ water drop (Fig 3A). The shaft of soil particles suppresses the sound. The local maximum for 9th water drop may result from the lack of the repeatability of the place in which water drops were placed. In other words the most likely the 9^th^ water drop hit the shaft–not the bottom of the crater.

The decreasing sound wave energy for the highest water content (0.1 kPa) of *Endogleyic Umbrisol* after the sixth water drop can be explained by the creation of the craters. (Fig 3A). The shaft of soil particles suppresses the sound. The local maximum for the ninth water drop may result from the lack of the repeatability of the place where the water drops fall. In other words, it is more likely that the water drop hit the shaft and not the bottom of the crater.

### The influence of the soil initial pressure head on sound wave energy

To consider the influence of the initial moisture content on the sound that result from the water drop hitting the soil, the graphs in Fig 2 should be analysed. For *Fluvic Endogleyic Cambisol* (Fig 2B) and *Haplic Chernozem* (Fig 2C) the highest values of sound pressure (and sound wave energy) were obtained for the wettest soil (pressure head 0.1 kPa). The values for 0.1 kPa were significantly different from other moisture levels (S3 Table). It is easy to find an explanation for this; when the soil is close to saturation at water drop hitting the surface is not able to penetrate into the deeper soil layers. A mini-pool forms on the surface and the next water drop hits the thin water layer, not the soil. Hence, there is a noise from the splashing of water.

The apparent negation of such an application is that for *Endogleyic Umbrisol*, the highest value of sound pressure was not for the highest water content (from the fourth water drop the highest sound pressure was for 3.16 kPa). This situation is caused by the absence of mini-pool on the soil’s surface. As discussed in the previous sub-section, this soil had the highest content of coarse fraction (sand). When the soil consists of many sand grains there are large pores between them [34]. In the investigated *Endogleyic Umbrisol*, over 90% of pores were bigger than 30 μm. In this case, the rate of water percolation is sufficiently high, since the large pores are not able to retain the water with capillary forces. Even when the sample is close to saturation the water flows downwards with gravity (cylinders containing the samples were secured by chiffon at the bottom, which is a porous material, some of the water seeped through the chiffon and drained away). Therefore, the sound pressure when water drop hits a surface is not connected with the water splash but rather with the deformation of soil surface. If so, it should not be expected that the sound pressure depends on the initial water content. The variability (in many cases statistically significant–S3 Table) observed in Fig 2A should be attributed to the heterogeneity and the lack of reproducibility of surface micro-relief.

The values of sound pressure for *Fluvic Endogleyic Cambisol* (Fig 2B) and *Haplic Chernozem* (Fig 2C) obtained for other than 0.1 kPa pressure heads were practically the same.

The last observation that should be pointed out is the lack of trends in the sound pressure (Fig 2) and, as a result in the energy of the sound wave between the successive water drops. In the vast majority of cases, the differences of the values between drops were not statistically significant (S3 Table).

## Conclusions

The use of measurements described in the work measurement system and testing in an anechoic chamber allowed the measurement of the sound pressure level (noise) and, on that basis, calculation of the resulting sound wave energy during the impact of drops on the soil surface.

It was found that the values of the sound pressure and sound wave energy were dependent on the particle size distribution of the soil, less dependent on the initial pressure head, and practically the same for subsequent water drops (from the first to the tenth drop). The sandier the soil, the higher the energy of the sound waves emitted after the water drop hit on the surface. Higher energy sound waves were also found for saturated soils. For the free impact on the soil surface of a water drop with a diameter of 4.279 ± 0.002 mm falling from a height of 1.5m with a kinetic energy of 0.491 mJ ± 0.012 mJ, the sound pressure level values ranged from 27 dB to 42 dB. The slightest noise impact was accompanied by drops of water on the soil surface of *Fluvic Endogleyic Cambisol* (an average of approx. 28.5 dB). The largest was found for the *Endogleyic Umbrisol* soil (an average of approx. 34 dB). In the case of the *Haplic Chernozem* soil, it amounted to an average of approx. 30.5 dB. On the basis of the calculations presented in this work, it was found that the energy emitted in the form of sound waves accompanying the impact was in the range of 0.14 to 5.26 μJ, which was between 0.03% and 1.07% of the energy of the incident drops.

## Supporting Information

S1 Fig Diagram showing the microphone arrangement. (PDF)

S1 Table **Sound pressure level at the time of a drop of water hitting the surface of the soils studied for four different initial pressure head for: a) *Endogleyic Umbrisol*; b) *Fluvic Endogleyic Cambisol*; c) *Haplic Chernozem***.
(PDF)

S2 Table **Sound wave energy for three different soils for initial pressure head: a) 0.1 kPa; b) 1 kPa; c) 3.16 kPa; d) 16 kPa**.
(PDF)

S3 Table **Results from median test for sound wave energy for four different initial pressure head for: a) *Endogleyic Umbrisol*; b) *Fluvic Endogleyic Cambisol*; c) *Haplic Chernozem* for α = 0.05.** Letters show the determination of the significance of differences for the energy of the sound wave evoked by the impact of a given drop at different initial moisture levels. Marks show the determination of the significance of differences for the energy of the sound wave evoked by consecutive drops at a given initial moisture level.
(PDF)
