# Supplementary material for: Sound Wave Energy Resulting from the Impact of Water Drops on the Soil Surface
Source: PLoS One. 2016 Jul 7;11(7):e0158472. doi: 10.1371/journal.pone.0158472 (PMC4936686; doi:10.1371/journal.pone.0158472)

SUPPORTING FIGURE S1 for

Sound wave energy resulting from the impact of water drops on the soil surface

Magdalena Ryżak, Andrzej Bieganowski, Tomasz Korbiel

S1 Figure. Diagram showing the microphone arrangement.

This scheme of measuring position is a way of illustration only and does not reflect the scale

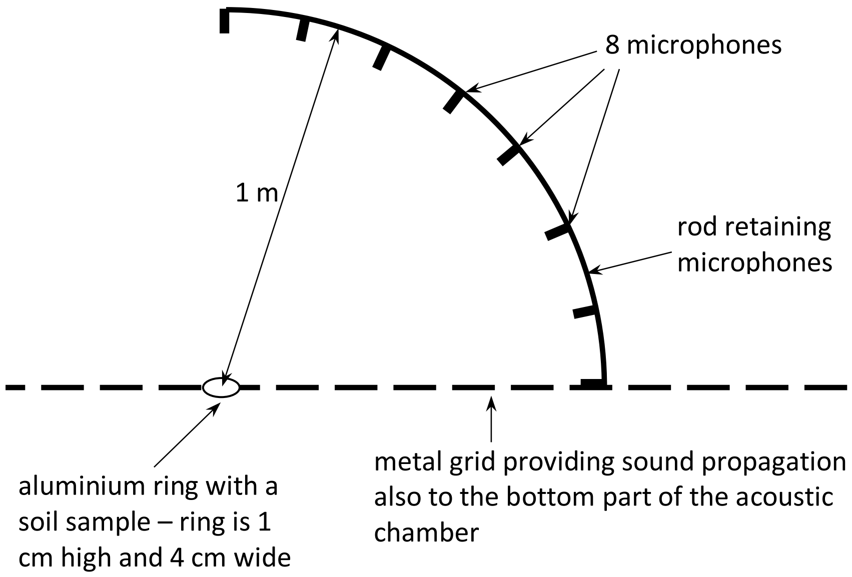

Supplement: S1 Fig — (PDF) [file pone.0158472.s001.pdf]
