# Supplementary material for: Sound Wave Energy Resulting from the Impact of Water Drops on the Soil Surface
Source: PLoS One. 2016 Jul 7;11(7):e0158472. doi: 10.1371/journal.pone.0158472 (PMC4936686; doi:10.1371/journal.pone.0158472)
Supplement: S1 Table — Sound pressure level at the time of a drop of water hitting the surface of the soils studied for four different initial pressure head for: a) Endogleyic Umbrisol; b) Fluvic Endogleyic Cambisol; c) Haplic Chernozem. (PDF) [file pone.0158472.s002.pdf]

SUPPORTING TABLE S1 for

Sound wave energy resulting from the impact of water drops on the soil surface

Magdalena Ryżak, Andrzej Bieganowski, Tomasz Korbiel

S1 Table. Sound pressure level at the time of a drop of water hitting the surface of the soils studied for four different initial pressure head for: a) Endogleyic Umbrisol; b) Fluvic Endogleyic Cambisol; c) Haplic Chernozem.

a)	Sound pressure level [dB] with SD							
Number of incident drops	0.1 kPa	1/2*SD	1 kPa	1/2*SD	3.16 kPa	1/2*SD	16 kPa	1/2*SD
1	33.2	1.5	29.4	0.7	32.4	1.9	30.0	0.9
2	35.6	0.8	30.2	1.1	34.0	2.3	32.2	1.3
3	36.3	1.2	31.2	1.1	32.9	3.0	32.6	1.9
4	36.8	1.1	31.5	1.3	38.2	1.4	33.1	1.8
5	37.4	1.1	32.1	1.3	39.3	1.1	33.0	1.8
6	37.6	1.3	32.5	1.7	39.8	2.2	33.0	2.1
7	36.5	1.7	33.1	1.0	40.5	2.2	33.8	1.6
8	36.3	1.1	32.6	1.5	39.3	2.4	32.3	1.9
9	37.0	1.1	33.3	1.4	41.7	0.6	31.7	2.2
10	36.4	0.8	34.8	1.2	42.2	0.7	33.4	2.7
b)	Sound pressure level [dB] with SD							
Number of incident drops	0.1 kPa	1/2*SD	1 kPa	1/2*SD	3.16 kPa	1/2*SD	16 kPa	1/2*SD
1	31.6	1.2	26.8	0.4	27.4	0.8	27.2	0.6
2	32.2	1.5	27.2	0.4	27.6	0.7	26.6	0.3
3	32.3	1.6	27.1	0.4	28.0	0.8	26.9	0.3
4	33.3	0.6	27.0	0.3	28.1	0.6	27.0	0.3
5	33.5	0.5	26.9	0.2	27.6	0.7	26.6	0.3
6	33.5	0.7	27.4	0.5	27.5	0.8	26.8	0.3
7	33.2	1.2	27.2	0.3	27.4	0.7	26.6	0.4
8	33.5	0.5	27.2	0.2	27.2	0.7	27.1	0.4
9	33.6	0.6	27.2	0.3	27.4	0.7	27.2	0.5
10	33.8	0.7	27.5	0.4	27.4	0.7	26.7	0.3
c)	Sound pressure level [dB] with SD							
Number of incident drops	0.1 kPa	1/2*SD	1 kPa	1/2*SD	3.16 kPa	1/2*SD	16 kPa	1/2*SD
1	35.4	1.2	29.4	1.3	28.2	0.8	27.6	0.2
2	34.4	0.5	29.6	1.0	28.6	0.7	27.6	0.3
3	33.8	0.6	29.7	1.0	28.9	0.8	28.0	0.3
4	34.4	0.6	30.1	0.7	29.1	0.6	28.1	0.3
5	34.8	0.6	30.5	1.0	29.1	0.7	28.4	0.3
6	34.1	1.3	30.5	0.6	29.1	0.8	28.7	0.3
7	34.8	0.9	30.8	0.7	29.2	0.7	28.8	0.3
8	34.2	1.0	30.9	0.7	29.3	0.7	29.0	0.4
9	34.1	0.6	30.9	0.8	29.7	0.7	29.2	0.3
10	34.2	1.1	31.0	0.7	29.7	0.7	29.0	0.3
