# Supplementary material for: Sound Wave Energy Resulting from the Impact of Water Drops on the Soil Surface
Source: PLoS One. 2016 Jul 7;11(7):e0158472. doi: 10.1371/journal.pone.0158472 (PMC4936686; doi:10.1371/journal.pone.0158472)
Supplement: S2 Table — Sound wave energy for three different soils for initial pressure head: a) 0.1 kPa; b) 1 kPa; c) 3.16 kPa; d) 16 kPa. (PDF) [file pone.0158472.s003.pdf]

SUPPORTING TABLE S2 for
Sound wave energy resulting from the impact of water drops on the soil surface

Magdalena Ryżak, Andrzej Bieganowski, Tomasz Korbiel

S2 Table. Sound wave energy for three different soils for initial pressure head: a) 0.1 kPa; b) 1 kPa; c) 3.16 kPa; d) 16 kPa.

a)	Sound wave energy [μJ] for different soils with SD					
Number of incident drops	Endogleyic Umbrisol	1/2*SD	Fluvic Endogleyic Cambisol	1/2*SD	Haplic Chernozem	1/2*SD
1	0.733	0.164	0.506	0.128	1.183	0.292
2	1.183	0.223	0.597	0.150	0.861	0.117
3	1.481	0.347	0.635	0.177	0.766	0.102
4	1.608	0.383	0.683	0.096	0.868	0.120
5	1.869	0.533	0.696	0.087	0.949	0.137
6	2.047	0.562	0.707	0.095	0.881	0.179
7	1.703	0.494	0.696	0.116	1.002	0.222
8	1.444	0.345	0.709	0.092	0.868	0.180
9	1.743	0.490	0.721	0.100	0.808	0.104
10	1.237	0.384	0.659	0.172	0.884	0.181
b)	Sound wave energy [μJ] for different soils with SD					
Number of incident drops	Endogleyic Umbrisol	1/2*SD	Fluvic Endogleyic Cambisol	1/2*SD	Haplic Chernozem	1/2*SD
1	0.281	0.058	0.147	0.015	0.310	0.095
2	0.370	0.125	0.160	0.014	0.316	0.099
3	0.447	0.116	0.157	0.015	0.318	0.094
4	0.505	0.145	0.155	0.011	0.328	0.057
5	0.585	0.187	0.150	0.008	0.370	0.080
6	0.681	0.211	0.173	0.029	0.357	0.056
7	0.703	0.186	0.161	0.012	0.384	0.070
8	0.664	0.195	0.160	0.009	0.393	0.064
9	0.778	0.256	0.160	0.011	0.403	0.083
10	0.979	0.319	0.176	0.020	0.406	0.076
c)	Sound wave energy [μJ] for different soils with SD					
Number of incident drops	Endogleyic Umbrisol	1/2*SD	Fluvic Endogleyic Cambisol	1/2*SD	Haplic Chernozem	1/2*SD
1	0.721	0.269	0.175	0.030	0.215	0.046
2	1.173	0.449	0.183	0.033	0.234	0.045
3	1.203	0.609	0.237	0.125	0.250	0.052
4	2.417	0.768	0.245	0.125	0.259	0.042
5	2.892	0.710	0.179	0.020	0.261	0.046
6	3.692	0.877	0.175	0.023	0.264	0.048
7	4.302	0.881	0.171	0.024	0.265	0.047
8	3.615	0.980	0.163	0.015	0.271	0.044

9	4.656	0.578	0.169	0.009	0.294	0.046
10	5.263	0.793	0.160	0.028	0.217	0.080
d)	Sound wave energy [μJ] for different soils with SD					
Number of incident drops	Endogleyic Umbrisol	1/2*SD	Fluvic Endogleyic Cambisol	1/2*SD	Haplic Chernozem	1/2*SD
1	0.325	0.058	0.166	0.028	0.176	0.009
2	0.597	0.188	0.142	0.010	0.178	0.012
3	0.831	0.511	0.149	0.009	0.195	0.011
4	0.877	0.449	0.154	0.013	0.198	0.015
5	0.825	0.337	0.140	0.010	0.212	0.015
6	0.927	0.436	0.149	0.011	0.228	0.017
7	0.935	0.334	0.142	0.014	0.231	0.017
8	0.703	0.263	0.159	0.014	0.246	0.021
9	0.735	0.420	0.166	0.028	0.255	0.018
10	1.294	0.788	0.145	0.009	0.245	0.016
