# Supplementary material for: Sound Wave Energy Resulting from the Impact of Water Drops on the Soil Surface
Source: PLoS One. 2016 Jul 7;11(7):e0158472. doi: 10.1371/journal.pone.0158472 (PMC4936686; doi:10.1371/journal.pone.0158472)
Supplement: S3 Table — Results from median test for sound wave energy for four different initial pressure head for: a) Endogleyic Umbrisol; b) Fluvic Endogleyic Cambisol; c) Haplic Chernozem for α = 0.05. Letters show the determination of the significance of differences for the energy of the sound wave evoked by the impact of a given drop at different initial moisture levels. Marks show the determination of the significance of differences for the energy of the sound wave evoked by consecutive drops at a given initial moisture level. (PDF) [file pone.0158472.s004.pdf]

SUPPORTING TABLE S3 for

**Sound wave energy resulting from the impact of water drops on the soil surface**

Magdalena Ryżak, Andrzej Bieganowski, Tomasz Korbiel

## S3 Table. Results from median test for sound wave energy for four different pressure head for: a) *Endogleyic Umbrisol*; b) *Fluvic Endogleyic Cambisol*; c) *Haplic Chernozem* for α=0.05.

Letters show the determination of the significance of differences for the energy of the sound wave evoked by the impact of a given drop at different initial moisture levels.

Marks show the determination of the significance of differences for the energy of the sound wave evoked by consecutive drops at a given initial moisture level.

a)

| | | Number of incident drops | | | | | | | | | | | | | | | | | | | |
|---|---|---|---|---|---|---|---|---|---|---|---|---|---|---|---|---|---|---|---|---|---|
| | | 1 | | 2 | | 3 | | 4 | | 5 | | 6 | | 7 | | 8 | | 9 | | 10 | |
| Sound wave energy | 0.1 kPa | a | * | a | + | a | + | a | + | a | + | a | + | a | + | a | + | a | + | a | + |
| | 1 kPa | b | * | b | * | b | * | b | * | b | * | b | * | b | * | b | * | b | * | a | * |
| | 3.16 kPa | b | * | c/a | */+ | b/a | */+/# | c | +/# | c | #/= | c | =/^ | c | ^/- | c | =/^/- | c | ^/- | b | - |
| | 16 kPa | c | * | c | + | b | + | d | + | d | + | d | + | d | + | d | + | d | + | c | +/* |

b)

| | | Number of incident drops | | | | | | | | | | | | | | | | | | | |
|---|---|---|---|---|---|---|---|---|---|---|---|---|---|---|---|---|---|---|---|---|---|
| | | 1 | | 2 | | 3 | | 4 | | 5 | | 6 | | 7 | | 8 | | 9 | | 10 | |
| Sound wave energy | 0.1 kPa | a | * | a | * | a | * | a | * | a | * | a | * | a | * | a | * | a | * | a | * |
| | 1 kPa | b | * | b | * | b | * | b | * | b | * | b | * | b | * | b | * | b | * | b | * |
| | 3.16 kPa | b | * | b | * | b | * | b | * | b | * | b | * | b/c | * | b | * | b | * | b | * |
| | 16 kPa | b | * | b | * | b | * | b | * | c | * | b | * | c | * | b | * | b | * | c | * |

c)

| | | Number of incident drops | | | | | | | | | | | | | | | | | | | |
|---|---|---|---|---|---|---|---|---|---|---|---|---|---|---|---|---|---|---|---|---|---|
| | | 1 | | 2 | | 3 | | 4 | | 5 | | 6 | | 7 | | 8 | | 9 | | 10 | |
| Sound wave energy | 0.1 kPa | a | * | a | * | a | * | a | * | a | * | a | * | a | * | a | * | a | * | a | * |
| | 1 kPa | b | */+ | b | * | b | * | b | */+ | b | + | b | + | b | + | b | + | b | + | b | + |
| | 3.16 kPa | b | * | b | */+ | b | */+ | b | */+ | b | */+ | b | */+ | c | */+ | b | */+ | b | + | c | + |
| | 16 kPa | b | * | b | * | b | * | b | */+ | b | +/# | b | +/# | d | +/# | b | # | b | # | c | # |
